# Genome assembly of six polyploid potato genomes

**DOI:** 10.1038/s41597-020-0428-4

**Published:** 2020-03-11

**Authors:** Maria Kyriakidou, Noelle L. Anglin, David Ellis, Helen H. Tai, Martina V. Strömvik

**Affiliations:** 1 Department of Plant Science, McGill University, 21111 Lakeshore Rd., Sainte-Anne-de-Bellevue, QC H9X3V9 Canada; 2 CIP-International Potato Center, Avenida La Molina 1895, Lima, 12 Peru; 3 Fredericton Research and Development Centre, Agriculture and Agri-Food Canada, PO Box 20280, 850 Lincoln Rd., Fredericton, NB E3B 4Z7 Canada

**Keywords:** Polyploidy in plants, Genome duplication

## Abstract

Genome assembly of polyploid plant genomes is a laborious task as they contain more than two copies of the genome, are often highly heterozygous with a high level of repetitive DNA. Next Generation genome sequencing data representing one Chilean and five Peruvian polyploid potato (*Solanum spp*.) landrace genomes was used to construct genome assemblies comprising five taxa. Third Generation sequencing data (Linked and Long-read data) was used to improve the assembly for one of the genomes. Native landraces are valuable genetic resources for traits such as disease and pest resistance, environmental tolerance and other qualities of interest such as nutrition and fiber for breeding programs. The need for conservation and enhanced understanding of genetic diversity of cultivated potato from South America is also crucial to North American and European cultivars. Here, we report draft genomes from six polyploid potato landraces representing five taxa, illustrating how Third Generation Sequencing can aid in assembling polyploid genomes.

## Abstract (summary)

**Table:** 

Measurement(s)	sequence_assembly
Technology Type(s)	sequence assembly process
Factor Type(s)	polyploid genome
Sample Characteristic - Organism	Solanum chaucha • Solanum x juzepczukii • Solanum tuberosum subsp. andigenum • Solanum tuberosum • Solanum x curtilobum
Sample Characteristic - Location	Peru • Chile

Machine-accessible metadata file describing the reported data: 10.6084/m9.figshare.11891199

## Background & Summary

Native potato species are distributed from the southwestern United States to Argentina^1^. The most commonly cultivated potato varieties are autotetraploids (2n = 4x = 48) with a base chromosome number of 12. However, cultivated potato landraces can range from diploids (2n = 2x = 24) to pentaploids (2n = 5x = 60)^2^ and wild potato species from the United States, Mexico and central America also include hexaploid species^3^. The potato genome is characterized by great heterozygosity, due likely to the fact that most of the diploid potato species are self-incompatible^2,4^.

A significant amount of work has previously been performed to aid the advance of potato genomics^5^. Currently, the publicly available potato reference genomes are from the doubled monoploid *Solanum tuberosum* Group *phureja* DM1-3^6^, the wild diploid *S. commersonii*^7^ and the diploid, inbred clone of *S. chacoense* - M6^8^. *S. tuberosum* is an autotetraploid, and evidence suggests the polyploid nature resulted through duplication events. Hence, a single reference genome cannot capture the great diversity found across different potato genomes, especially in the case of polyploids since they are more heterozygous than the diploids^9,10^. Improvement of current algorithms and of current sequencing technologies are fundamental to improving the assembly of polyploid genomes such as those found in diverse potato species^11^. Next Generation Sequencing (NGS) made a revolution in approaches to genome sequencing, due to reduced costs and faster sequencing compared with Sanger sequencing technology. However, NGS does have drawbacks, especially when sequencing polyploid genomes, where their short length can lead to misassemblies and extremely fragmented genome assemblies. The most recent evolution in the era of genome sequencing is the Third Generation (or Long-read) Sequencing (TGS) technologies, which can produce high quality genome assemblies with high resolution due to the longer length of the reads. TGS technologies can reduce the problem of assembling polyploid plant genomes^11^. Various complicated polyploid plant genomes have been sequenced with TGS technologies including *Chenopodium quinoa* (3x)^12^ and *Saccharum* sp (varying ploidy levels)^13^, *Fragaria x ananassa* (8x)^14^ and others.

Twelve potato genomes of various ploidy levels were recently sequenced^10^. These genomes, which were selected based on the Hawkes taxonomy^15^, in addition to the *S. commersonii* genome^7^ were compared to the two publicly available reference genomes *S. tuberosum* Group Phureja (DM1-3)^6^ and *S. chacoense* M6 clone^8^ for copy number variation (CNV) and SNP analyses. The study showed the great diversity across this panel of potato genomes and identified a number of CNVs in genes implicated in disease resistance and stress, among other processes.

In the present study, we have focused on assembling the reads for the six polyploid genomes from the previously sequenced cultivated potato landraces covering five taxa (based on)^10,15^: *Solanum chaucha* (3x: CHA), *S. juzepczukii* (3x: JUZ), two genomes of *S. tuberosum subsp. andigena* (4x: ADG1 and ADG2), *S. tuberosum subsp. tuberosum* (4x: TBR) and *S. curtilobum* (5x: CUR). One of the genomes, ADG1 – a tetraploid, is assembled with TGS and has therefore a higher quality assembly, while NGS data is used for the others.

## Methods

### Genomic data

Genome Illumina PE sequence data was generated for the six polyploid genomes: *Solanum chaucha* (3x: CHA – CIP 707129 doi:10.18730/CS5*), *S. juzepczukii* (3x: JUZ – CIP 706050 doi:10.18730/C09D), two genomes of *S. tuberosum subsp. andigena* (4x: ADG1 - CIP 700921 doi:10.18730/91RP; ADG2 - CIP 702853 doi:10.18730/9GB8), *S. tuberosum subsp. tuberosum* (4x: TBR – CIP 705053 doi:10.18730/B3MN) and *S. curtilobum* (5x: CUR – CIP 702937 doi:10.18730/9H1Y), from the *in vitro* potato germplasm collection at the International Potato Center (CIP) in Lima, Peru^10^. Genomic DNA was extracted and sequenced using an Illumina HiSeq sequencer (Illumina, Inc.) in paired-end mode (2 × 150 bp) as described^10^. The genome of ADG1 was also sequenced (~50x) with PacBio’s Single Molecule RS II system technology^16^ and with 10X Genomics’ GemCode technology (~134x)^17^ by Novogene^TM^.

### Determining the whole genome heterozygosity

Trimmed sequencing reads were used for the calculation of the percentage of heterozygosity in the genomes^10^. For this, jellyfish v2.2.10^18^ was first used to compute the histogram of the k-mer frequencies. The final k-mer count histogram per genome was used within the GenomeScope 2.0 online platform^19^.

### *De novo* genome assemblies

#### ADG1 assembly

Because of the availability of Linked and Long Reads, the genome of ADG1 genome was assembled following a hybrid-read method. Multiple approaches were tried but the best assembly possible was obtained using a combination of Long and Linked Reads with Canu^20^ and Supernova^TM^ assemblers^17^. For the following analyses, pseudohap1 was used as suggested in the genome assembly of *Capsicum annuum*^21^ with 10X Genomics reads. Μoreover, the Long Reads from PacBio were assembled with Canu v1.5 assembler^20^, then Tigmint v0.9^22^ was used to correct PacBio misassemblies using the parameters from 10X Genomics. The contigs were assembled into scaffolds with ARCS v1.0.2^23^. The final genome assembly was aligned to the DM1-3 v4.04^24^, and BUSCO v3.2.0^25^ and QUAST^26^ v5.0.0 were used for the evaluation of the assembly. Transposable elements and repeat masking was performed with RepeatModeler v1.0.11^27^ and RepeatMasker v4.0.7^28^.

#### CHA, JUZ, ADG2, TBR, and CUR assemblies

The Illumina PE reads of the CHA, JUZ, ADG2, TBR, and CUR genomes were assembled using MaSuRCA v3.2.4^29^. Redundant contigs were removed from the assembly using CD-HIT v4.8.1^30^ with identity > 90%. The resulting assemblies were evaluated using BUSCO v3.2.0^25^ and QUAST^26^ v5.0.0. From all the genome assemblies (ADG1, ADG2, TBR, JUZ, CHA and CUR), any mitochondrial and chloroplast genome has been removed, along with the contigs with length smaller than 200 bp.

## Data Records

The reads data is available as BioProject PRJNA556263 (SRA accessions SRR10237766, SRR10242927, SRR10248510 – SRR10248515^31–38^) at NCBI. The final genome assemblies are deposited into NCBI Assembly database under the following Accession Numbers: GCA_009849705.1, GCA_009849725.1, GCA_009849745.1, GCA_009849685.1, GCA_009849625.1, and GCA_009849645.1^39–44^.

## Technical Validation

### Quality of the sequenced genomes – whole genome heterozygosity

The read coverage ranged between ~36 X in the pentaploid CUR and 44.4 X in the triploid CHA for the Illumina reads (Table 1). The read coverage for the ADG1 genome was calculated with linked and long reads and it had an average read coverage of 50x (Table 1). The k-mer frequencies were calculated for each of the genomes (Supplementary Figs. 1–6). In general, there is a tendency towards bimodal distributions. In addition, the heterozygosity of the genomes ranges between 3.52% (in ADG1) and 12.02% (in CUR) (Table 1). The heterozygosity is confirmed by the k-mer frequency of the genomes and the bimodal distributions, which has previously been reported for polyploid genomes^45^.

**Table 1:** Assembled genomes, along with the technologies used for sequencing and their references.

*Solanum* full taxon name^a^	Ploidy	Accession	Code Name	Technology	NCBI record	Coverage (X)	Heterozygosity %^10^
*Solanum chaucha*	3x	CIP 707129 doi:10.18730/CS5*	CHA	Illumina PE	^43^	44.4	3.7
*S. juzepczukii*	3x	CIP 706050 doi:10.18730/C09D	JUZ	Illumina PE	^42^	37.7	7.3
*S. tuberosum subsp. andigena*	4x	CIP 700921 doi:10.18730/91RP	ADG1	10X Genomics PacBio	^39^	50	3.52
*S. tuberosum subsp. andigena*	4x	CIP 702853 doi:10.18730/9GB8	ADG2	Illumina PE	^40^	43.6	7.75
*S. tuberosum subsp. tuberosum*	4x	CIP 705053 doi:10.18730/B3MN	TBR	Illumina PE	^41^	40.3	8.43
*S. curtilobum*	5x	CIP 702937 doi:10.18730/9H1Y	CUR	Illumina PE	^44^	35.8	12.02

^a^Taxonomy based on^15^.

### Genome assembly of ADG1

A draft genome assembly of the *S. tuberosum subsp. andigena* (CIP 700921 doi:10.18730/91RP) – ADG1 was generated using a hybrid assembly approach of Third Generation Sequencing Data: Linked and Long reads (Table 1). This methodology was applied as it was previously tested in the group and was found to be the best approach for the data available. The initial assembly contains 87,194 contigs, with an N50 of 62,124 bp (Table 2). The final assembly, after removing redundancy, consists of 35,961 scaffolds and an N50 of 122,016 bp (Table 2). The genome size was estimated with a 10X Genomics Chromium library at 896.84 Mb, which is close to the size of other potato genomes^6–8^. The size of the assembly including only scaffolds longer than 10 kb, reaches 713.51 Mb. For the evaluation of the genome completeness of ADG1, BUSCO^25^ was used, finding 85.8% of BUSCO’s core Plantae ortholog genes present in the assembly and another 8.5% present as partial sequences (C:85.8%[S:76.3%, D:9.5%], F:8.5%, M:5.7%, n:1375).

**Table 2:** Genome assembly statistics of the ADG1, ADG2, TBR, JUZ and CUR genomes. (Values in parentheses are before removing redundant contigs).

Quality metric	ADG1	ADG2	TBR	JUZ	CHA	CUR
# of contigs	35,961	310,723	1,272,956	249,222	259,834	578,826
	(*87,194*)	*(*826,888*)*	*(4,334,576)*	*(692,839)*	*(608,922)*	*(1,348,978)*
Contigs > 1000 bp	35,744	248,064	271,542	194,864	194,390	364,379
	—	*(456,177)*	*(271,558)*	*(436,731)*	*(344,939)*	*(657,215)*
Length of assembly (Mb)	841.4	991.1	1,032	1,002	790.3	1,208
	*(842.0)*	*(1,611)*	*(1,598)*	*(1,800)*	*(1,251)*	*(2,067)*
GC %	34.83	34.84	35.69	35.41	35.32	36.27
	—	(*35.2)*	*(36.05)*	*(35.63)*	*(35.88)*	*(36.7)*
Largest contig length (Kb)	3,384	102	73	112	105	118
Contig N50	122,016	4,721	1,193	7,359	4,795	3,176
	*(62,124)*	*(3,154)*	*(267)*	*(4,598)*	*(3,335)*	*(2,221)*
Contig L50	1,312	59,398	207,326	84,278	42,633	109,841
% BUSCO present genes	85.8	53	18.8	58.6	54.1	45.8
% BUSCO partial genes	8.5	28.4	35.6	27.2	24.7	34.6
% BUSCO duplicated genes	9.5	5.7	3.5	6.9	4.1	6

To identify and mask the repetitive elements in the ADG1 assembly, RepeatModeler^27^ was used to construct a repetitive library, followed by RepeatMasker^28^. About 60% of the assembly was masked. Table 3 shows the repetitive content of the ADG1 genome.

**Table 3:** Repeat Content of the ADG1 assembly. Data generated with RepeatMasker^28^.

Element	Number of Elements	Length Occupied (bp)	Percentage of sequence
LINEs	43,676	18,473,203	1.70
LTR elements	219,424	261,037,853	23.98
DNA elements	28,736	14,413,138	1.32
Simple repeats	171,025	9,049,100	0.83
Low complexity	36,517	2,254,450	0.21
Unclassified	1,109,924	333,262,813	30.61
Total bases masked		515,341,644	60.20

### Genome assembly of CHA, JUZ, ADG2, TBR and CUR genomes

The initial genome assemblies were longer than the size of other reported potato genomes^6–8^ (Table 2). For instance, the CUR genome assembly was about 2.4 times longer than the potato reference genomes, which had genome sizes equal to 884.1 Mb (DM1-3), 830 Mb (*S. commersonii*) and 825.7 Mb (*S. chacoense*). The JUZ, ADG2, TBR genome assemblies were at least double the length the reference genomes, while CHA was shorter than the rest of the polyploid genomes (Table 2). These differences are likely due to the high heterozygosity in these polyploid genomes. Therefore CD-HIT^30^ was used to remove the redundant contigs that were present in each of the assemblies. After removing the redundant contigs from the genomes, the final contig number was reduced to almost a third of the initial number, while the genome size is 0.66% smaller compared to the initial assembly (Table 2). The assembly statistics improved after removing the redundant contigs.

**Fig. 1:**
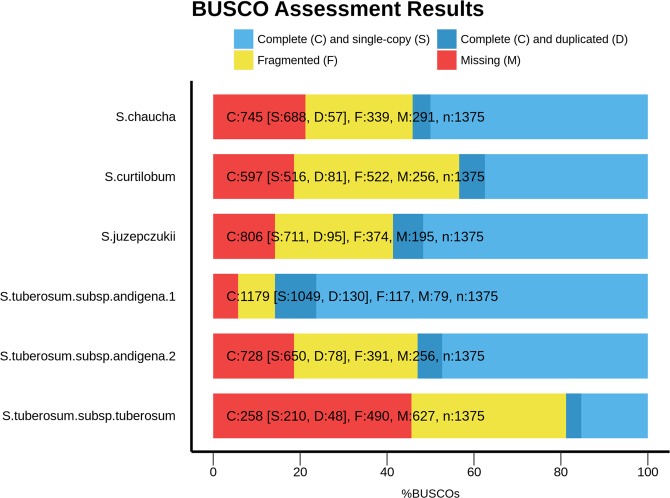
Bar chart with summary assessments for the proportion of genes present in six assembled polyploid potato (*Solanum* sp.) genomes. The summary assessment shows: Light blue shows the % of complete and single copy genes, the darker blue shows % complete and duplicated genes, the yellow shows the % of fragmented genes and finally the red shows the % of missing genes in the assemblies.

Even though the removal of the redundant contigs improved the genome assemblies, the assemblies are still very heterozygous and very fragmented (Table 2). Based on the gene content, the TBR assembly is the most fragmented. Figure 1 shows that presence of BUSCO’s core Plantae ortholog genes in TBR almost reached 18.8%, while the majority (35.6%) are partial genes. For the rest of the genomes, the amount of orthologous genes did not exceed 58.6% (Fig. 1, Table 2; JUZ), with an average amount of fragmented genes at 27.7%. The quality of the Illumina PE genome assemblies was similar among the genomes, with TBR being the exception.

### Comparison of the genome assemblies of ADG1 and ADG2

Table 2 shows that the genome assembly of ADG1 using Linked and Long reads yielded 35,961 contigs, compared with the ADG2 assembly using only Illumina reads that yielded 310,723 contigs – almost one order of magnitude difference. Moreover, almost all the contigs of ADG1 are greater than 1,000 bp in length, while only 248,064 contigs (~80%) of the ADG2 have lengths greater than 1,000 bp. The N50 for ADG1 is 25.8 times larger than that of ADG2. Finally, in ADG1 85.8% of the BUSCO genes were present, in contrast to ADG2, where only 53% of BUSCO genes were detected. The GC% content was very close for both genomes; 34.83% and 34.84% for ADG1 and ADG2, respectively.

### Comparison of the genome assemblies of ADG1 and ADG2, TBR, JUZ, CHA, and CUR

As shown in Table 2, the largest genome assembly is that of the pentaploid CUR genome (1.2 Gb), while the shortest is the triploid CHA genome (790.4 Mb). The TBR assembly was the most fragmented (1,272,956 contigs) compared to the rest of the genomes. Additionally, in TBR, only 21.3% (271,542) of the total number of contigs have length more than 1,000 bp, while 78.16% (194,864) of the JUZ’s contigs and all the contigs of ADG1 are larger than 1,000 bp. The GC% content ranged between 34.83% (in ADG1) and 36.27% (in CUR). ADG1 had the largest contig (~3.4 Mb), followed by CUR (117.7 kb) and JUZ (112 kb). The N50 is dramatically improved in the ADG1 compared to the others. TBR has the smallest N50 (1,193), showing once again the very fragmented assembly due to the high heterozygosity of this genome. Finally, all the genomes had more than 43% of BUSCO’s genes present, except TBR, in which only 18.8% of the total BUSCO genes were found.

## Usage Notes

### Highly fragmented genome assemblies due to the heterozygous nature and repetitiveness of the polyploid potato genomes

The high ploidy level can lead to higher heterozygosity, causing difficulties in haplotype identification in assemblies without Long range or Long read data^11^. In the current study, the CHA, JUZ, TBR, and CUR assembled polyploid genomes are highly fragmented, while the ADG1 assembly, which included Long Range data, resulted in the construction of a less fragmented genome, less redundant and with fewer contigs. This demonstrates the benefit and need for Long range data for complex genomes. Additionally, there has been innovation in novel assembly algorithms and new assembly strategies using Long range data for the genome assembly of polyploid genomes^11^. Moreover, the repetitiveness of the potato genome makes its assembly even more difficult. It appears that 60.2% of the ADG1 genome accounts for repetitive sequences, which is also in agreement with previous contents of repetitive sequences in other potato species; 62.2% in the *S. tuberosum* DM1-3 genome and 60.7% in the M6 clone of the *S. chacoense*^8^.

Among the six assembled genomes, the triploid CHA is the shortest. In previous studies using copy number variation analysis and SNP detection analysis of this genome (compared to the DM1-3 genome), it appears less heterozygous than JUZ, which is also a triploid, but also less heterozygous than the rest of the polyploids^10,46^.

The most challenging genome to assemble was the tetraploid TBR and not the pentaploid CUR, as would have been expected. It may be that the greater heterozygosity in TBR lead to it being the most fragmented genome assembly. This is supported by a previous study using the Infinium 12 K V2 Potato Array in a subset of the CIP potato collection – TBR were among the species with the highest amount of admixture^46^. Even in relation to other tetraploids, TBR appears to be the most heterozygous when compared to the DM1-3 v4.04 reference^10^. High levels of heterozygosity were observed from the sequencing data of the cultivated clones in the study. The clonal propagation of potato over thousands of years limited genetic recombination and led to high levels of heterozygosity. Polyploidy and self-incompatibility may also have contributed.

The genome assembly of plant genomes, and especially polyploid plant genomes, is very complex and challenging. The genome assemblies of two triploid (3x), three tetraploid (4x) and one pentaploid (5x) potato were constructed. Even though the majority of the assemblies are fragmented, these genomes provide a great resource to enhance potato breeding. It is known that the polyploid genomes contain more genes, hence these potato genomes can be explored for their genetic content. Moreover, as predicted, the availability of Third Generation Sequencing data greatly reduces the genome assembly problem.

## Supplementary information

Supplementary figures
