# Supplementary material for: Genome assembly of six polyploid potato genomes
Source: Sci Data. 2020 Mar 11;7:88. doi: 10.1038/s41597-020-0428-4 (PMC7066127; doi:10.1038/s41597-020-0428-4)
Supplement: Supplementary file 1 — Supplementary figures [file 41597_2020_428_MOESM1_ESM.pdf]

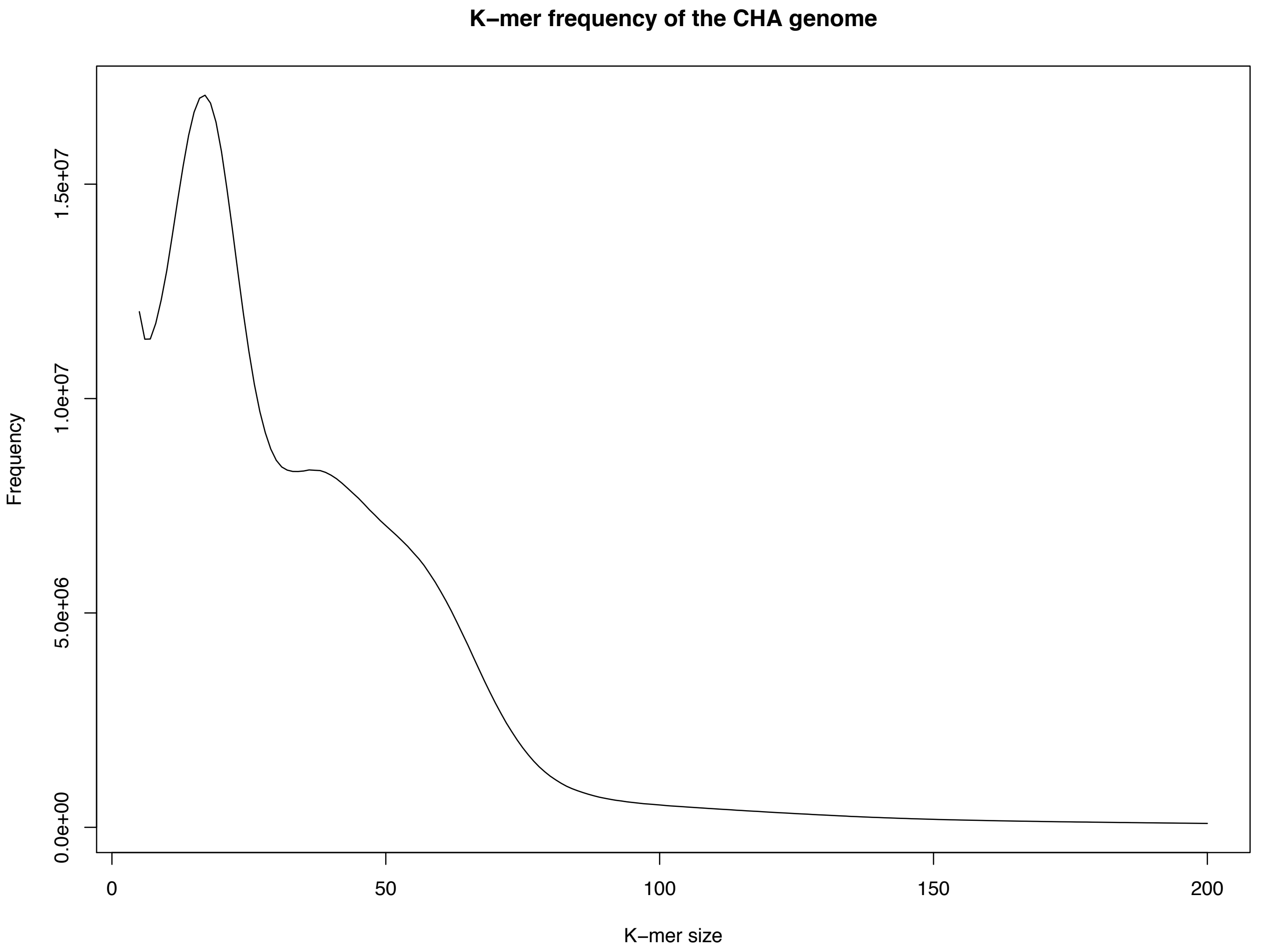

Supplementary Figure 1: The k-mer frequency of the CHA genome. The increased heterozygosity of the genome is validated by the tendency towards bimodal distribution of the k-mer frequency.

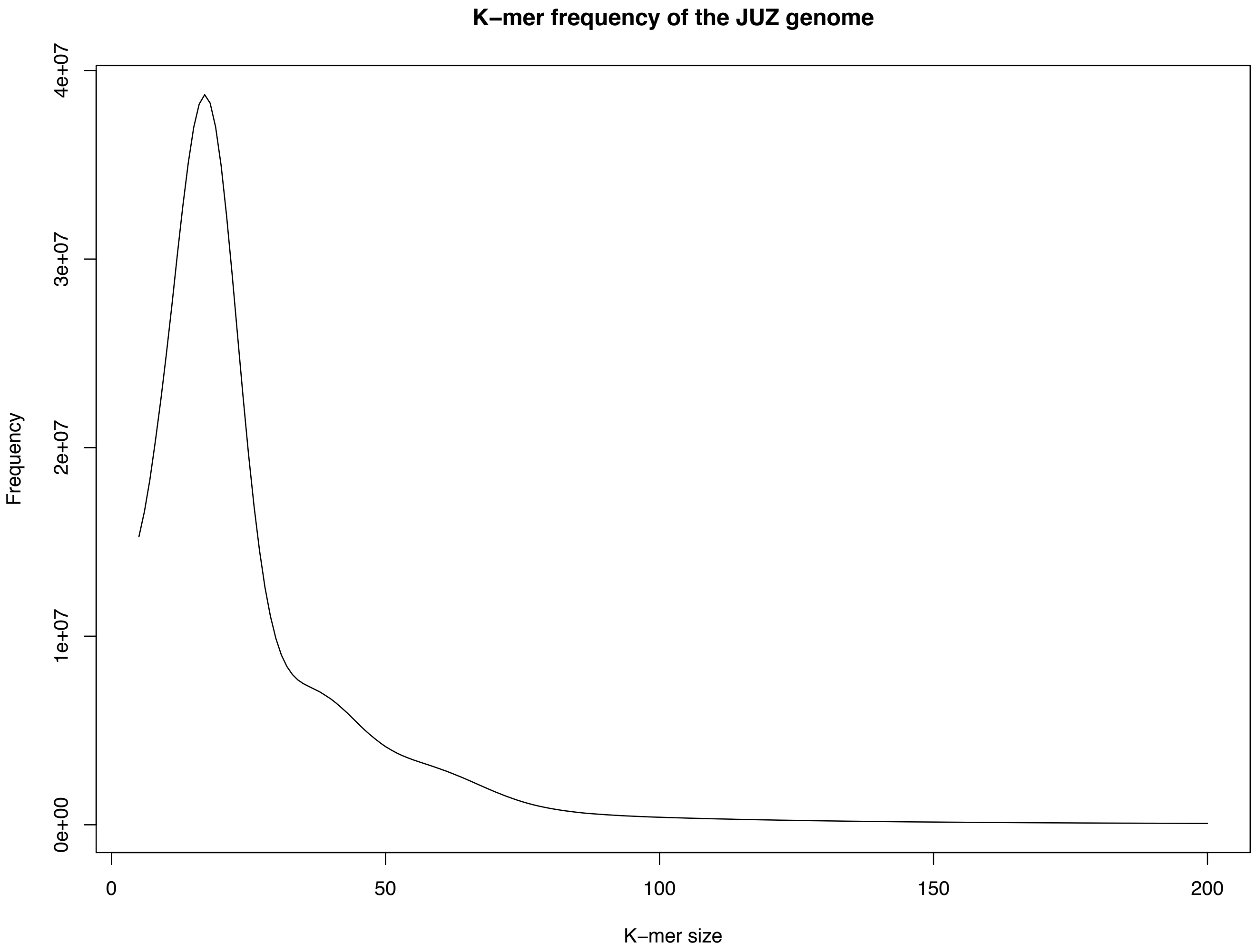

Supplementary Figure 2: The k-mer frequency of the JUZ genome. The increased heterozygosity of the genome is validated by the tendency towards bimodal distribution of the k-mer frequency.

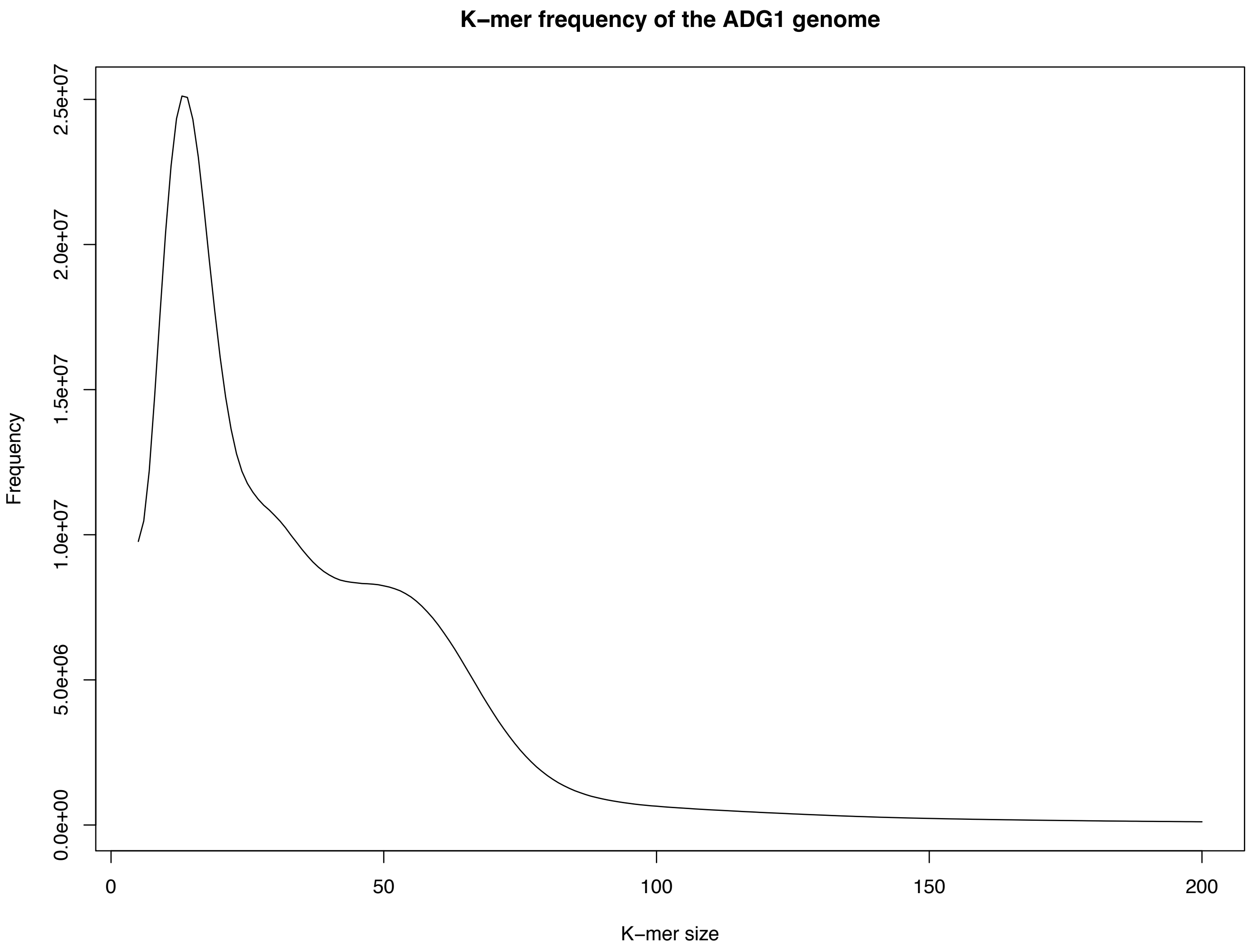

Supplementary Figure 3: The k-mer frequency of the ADG1 genome. The increased heterozygosity of the genome is validated by the tendency towards bimodal distribution of the k-mer frequency.

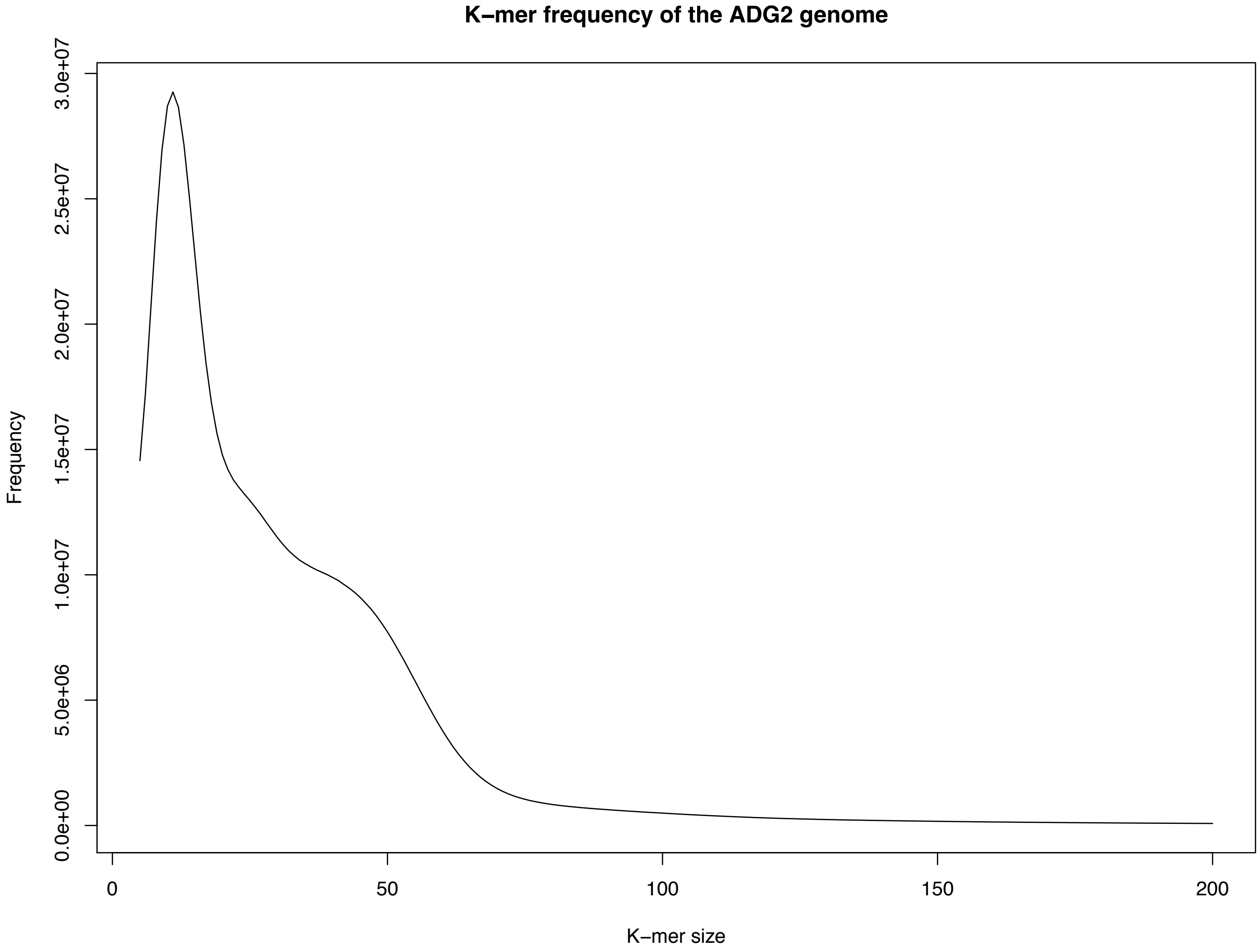

Supplementary Figure 4: The k-mer frequency of the ADG2 genome. The increased heterozygosity of the genome is validated by the tendency towards bimodal distribution of the k-mer frequency.

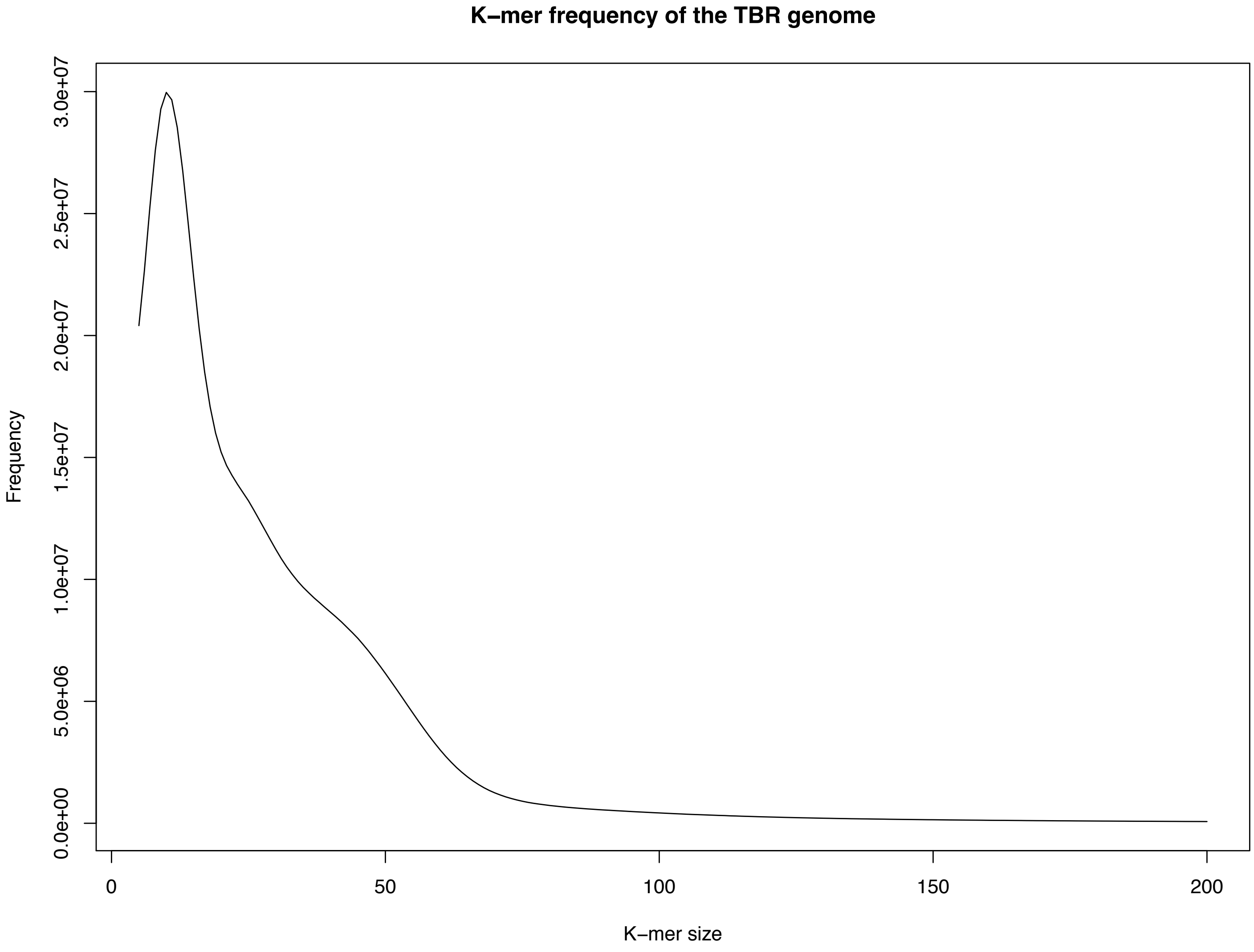

Supplementary Figure 5: The k-mer frequency of the TBR genome. The increased heterozygosity of the genome is validated by the tendency towards bimodal distribution of the k-mer frequency.

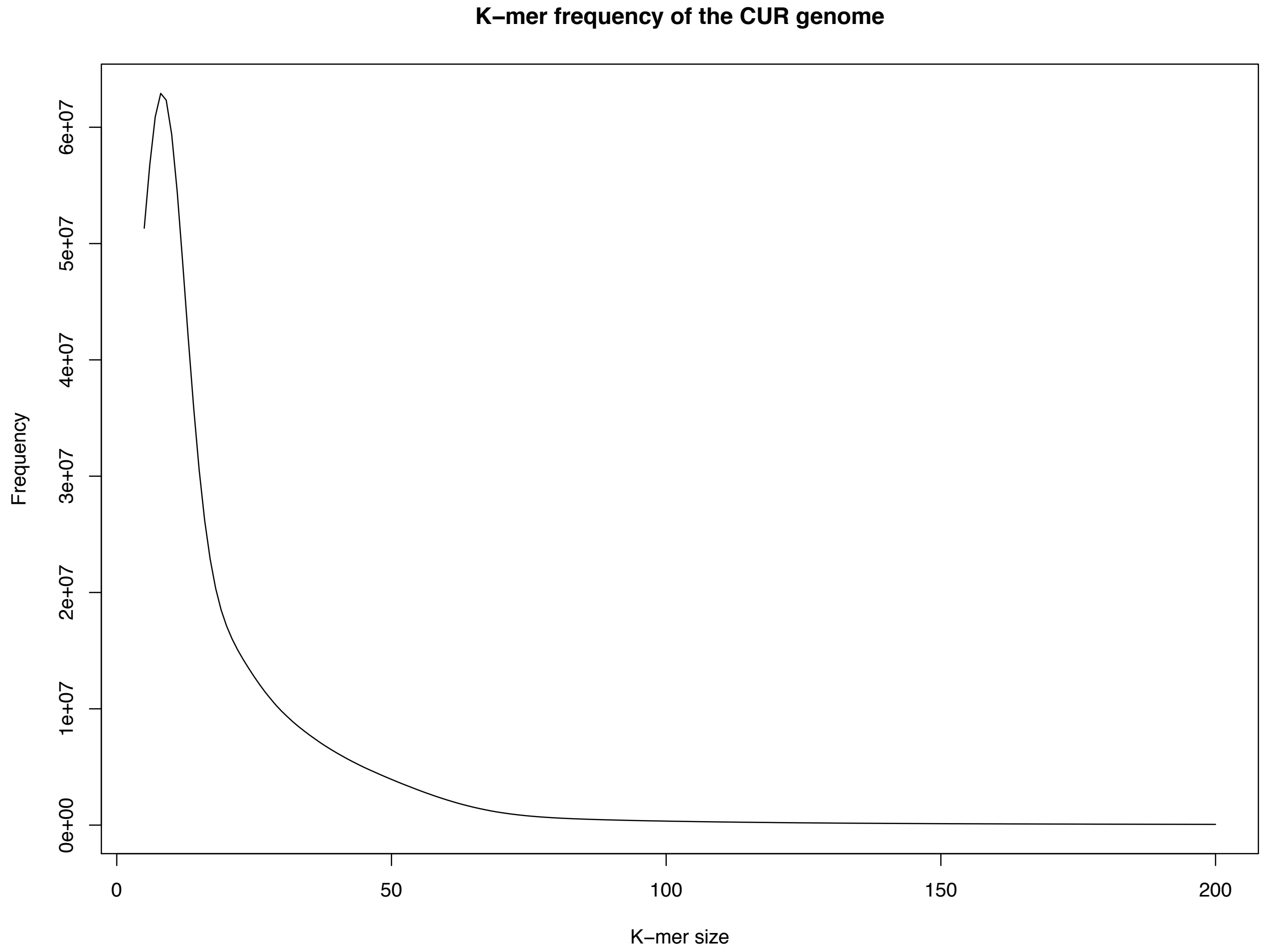

Supplementary Figure 6: The k-mer frequency of the CUR genome. The increased heterozygosity of the genome is validated by the tendency towards bimodal distribution of the k-mer frequency.
